# On the Inexpediency and Impracticability of Teaching Dental Surgery in Medical Colleges

**Published:** 1851-07

**Authors:** Elisha Townsend

**Affiliations:** Philadelphia.


ARTICLE II.

### *On the Inexpediency and Impracticability of Teaching Dental Surgery in Medical Colleges.* By ELISHA TOWNSEND, D. D. S., Philadelphia.

DR. E. B. GARDETTE, a practicing dentist of this city, has a memorial or address in the April number of, "The American Journal of the Medical Sciences," to the trustees and faculties of the medical schools of the United States, upon the propriety and advantages of establishing an adjunct professorship to the chair of surgery, in which the specialty of dental surgery may be taught to the medical students attending those institutions.

The system of public instruction in medicine and surgery, now in use in this country is by no means settled or satisfactory to the profession; and dental colleges have so recently come into existence, and are still so few in number, that their organization and method have not yet had the benefit of a large and varied experience, nor, perhaps, have they been very thoroughly considered even in theory. Dr. Gardette's proposition brings the order and policy of both into discussion, together as one scheme. The subject is of very great and pressing importance to dental science, especially, for its highest interests are pivoted upon the policy of public teaching, now soon to be settled definitively by its friends in this country. In the memorial these positions are the principal ones taken. The existence of such a chair in the medical colleges would be generally useful to those, who, to the practice of medicine and surgery may be compelled to add, also, the practice of some branch of dental surgery; and to those among the students in attendance at such colleges, who design to embrace dentistry as their proper profession. The plan is recommended to meet these wants, and urged as a duty and interest of the medical schools. The duty is put upon the ground of the equal importance of dentistry, with the other branches of general surgery, for which such provision is now made. The dental colleges established within a few years are alluded to, "without examining their history or influence upon the profession," and without investigating the capability or necessity of such distinct institutions for dental education, but to express a regret, and ground a reproach, against the medical schools, for forcing them into existence by the "unjust exclusion of this branch" from the general course. The "singling out of the profession of dentist from among the specialties of surgery and forcing it into an attitude of independence," is attributed to an "inexplicable unkindness." On the point of independent dental colleges generally, the writer contents himself with saying, that the principle would involve the "dismembering of a great family," the injury alleged being the loss to the several branches of the *esprit du corps ;* not stated argumentatively, but, in the author's words, as an "expression of opinion" merely. The *interest* of the medical schools in the adoption of the plan, is intimated in an inference that "the students who *now* attend the dental schools would, in this event, be added to the number of matriculates in the medical classes of the country." Considering the doctor's article as it bears upon the question of separate or blended systems of public teaching, I need not notice such general remarks upon the topic as equally concern both, and with this qualification, only, I believe, I have here reproduced the substance and the strength of Dr. G's points, and his arguments in their support. I will now notice what he does *not* examine, discuss or settle, giving my own thoughts freely upon the ground taken, and neglected, and upon related topics generally.

Our best medical colleges have now seven departments of instruction, and as many separate chairs and professors. The adjunct professorship of this plan, would be an eighth, as much as if it were on geology or comparative anatomy, instead of a separate specialty of surgery. But Dr. G. has not once glanced at the practicability, the possibility of adding another to the over crowded divisions of general medicine. The question first to be settled, is, can it be done—is there room for it? Does he propose to add one more lecture in the day, every day, or any day in the week, to the number now given in our schools? or, does he propose to dock the other seven branches of their fair proportions, to make a place for this one? The doctor has not been attentive enough to the practicability of his scheme, to consider the details at all. The answer of the professors in the several faculties who are most capable of settling it, would be, with one voice, that the principal branches now, have not sufficient time allowed them, to get through with their proper duties, and that the students were already crammed and worked beyond the point most profitable within the terms allowed for their attendance upon the lectures. Dissection, reading and rest, are now too heavily taxed by the hours required in the lecture rooms. In a word, that the existing system is pressing for longer sessions, or more of them, and that there is little or no hope of relief and amendment in this direction. This being the state of things, I may remark here, that the medical schools are fully vindicated against the charge of "inexplicable unkindness," in the exclusion of dentistry from their catalogue of subjects, though the neglect may have happened in fact from other causes, such as the fact, that, years ago, there was nobody to teach it scientifically, and, if it be really impracticable now, or if it can be much *better* taught in separate establishments, their justification for continuous exclusion is complete. But this last point it is the proper province of the dentists to settle. Dr. Gardette has given his opinion and project, and it is but due to both sides of the question to say, that it could not find an abler advocate, or afford a better defence than he can give it, and I conclude that is owing to an inherent weakness in the proposition itself that the doctor totally neglects to show the capability of an adjunct professorship, a single chair in a crowd of eight, to teach dentistry successfully. Any tolerable analysis of the matters embraced in a comprehensive scheme of dental education would, in the mere statement, look much more like a programme for a faculty than for a single teacher. Such an adjunct chair in a medical college, in fact, makes room for nothing that would not be as acceptable and useful to the pupils in books, or in the form of lectures, printed, as by delivery from the desk. Indeed, if dentistry could find room in a chink so narrow as this, it is not so much of a science, or of a profession, for exclusive study and practice, as we had certainly, and somewhat proudly thought it. But the argument for the proposition is unsatisfactory, not only from its inattention to primary and essential points, conditions precedent, as the lawyer would say, without which neither the reasoning, nor the subject matter can get forward, but it is feeble also in the looseness of its reasoning, wherever that is vouchsafed; for instance, the establishment of separate schools for dental surgery, is huddled into a heap with distinct and separate schools for the surgery of the eye, the ear, &c. Now the word *specialty*, though well enough applied to each, does not establish a resemblance sufficient for the purpose of argument, for, the first question in classifying things is, are they alike in all the circumstances concerned? clearly they are not. To spare the labor of more abstruse inquiry, I will rest it upon such open and obvious points as these, in full confidence of their sufficiency. Dentistry and general surgery are inevitably distinct professions, both in acquirement and practice. No surgeon of eminence, though he does practice, more or less, in all the branches of his science, now taught in the schools, ever performs any dental operations, except, perhaps, the occasional extraction of a tooth. Beyond this, he is as certain, in his need, to call upon a dentist, as upon a lawyer, for their respective services, and for the same reason, his own incompetency. The science of dentistry cannot be picked out of a few general physiological and pathological principles that lie within his reach; nor does its practice lie within the compass of a few prescriptions and general directions. And, on the other hand, nobody ever heard of a dentist undertaking the whole range of surgery, not, I presume to say, because of his incompetency, for, among the twenty-five hundred in the country spoken of by Dr. G., there must be at least the average of education and ability in a considerable number of them; but, because every good dentist really has his hands full of one whole profession already. Besides the surgery proper of our profession, which is various, nice and difficult, there are mechanical and artistic departments, which need the education of the eye and hand, and the cultivation of a good taste, or, in the language of Dr. Hullihen, quoted by Dr. G. for another purpose, "It required great mechanical skill, which they (medical men and surgeons) had not received and knew not where to acquire. * * * * * * The necessities of the community cried abroad to them for help, which they could not bestow." This is spoken of an earlier period, but it describes the present with equal accuracy. To this may be added the frequent occasions for consultations between the physician, the surgeon, and the dentist, spoken of by Dr. G. as another evidence of the distinctness which we allege and of the practical impossibility of blending the professions even in theory, for the surgeon, it is presumed calls upon the dentist for something more than the mechanical services he can render—this would *not* be *consultation*, but dictation. In fact the surgeon needs for his instruction the *better* knowledge and experience of the dentist in the special range of his profession, than the general practitioners can possibly have. Every where the evidence is abundant that the two avocations are more than one man's work, and that the two sciences are more than a sufficient task for the capacity of a single student and *cultivator*. Even Dr. G. himself, unconsciously concedes one point in his own proposition. He suggests, because he feels the necessity of it, at least one separate chair for our branch, and does not propose to run it in upon general surgery, as another specialty, entitled to an equal place, and capable of equally easy accomodation with lithotomy, opthalmia, surgery, &c. The whole argument raised upon the resemblance goes to pieces when such a difference as this is felt and shown; for if dentistry be that much more extensive as to require its *own* chair, it *may* be so much so as to require two, three, four, or five professorships, a special anatomy and chemistry of its own, a dispensary, and the entire apparatus of a college. A moment since I said that general surgery and dentistry is more than a sufficient task for the capacity of a single student and cultivator. I did not mean that a surgeon of ability could not acquire what is known of dental science in addition to his own profession proper, any more than I intended to deny to Dr. Gardette, or Dr. Hullihen, or any other eminent dentist, the capability of adding the science of general surgery to his own professional learning. It was with reference to the demand which Dentistry now makes upon its foremost professors for liberal enlargement, greater development, complementary discovery in the science and practice, along with an adapted system of education for its pupils, now pressingly required, that I would set apart the best talents we can command for its *exclusive cultivation*, and deem the task a full one for any rank of mind. And it is in this behalf above all others, that I would give our science the benefit of the utmost subdivision in its method of study and instruction, which unites convenience with utility, and would provide its principal men, with all the conditions, appliances and motives, for the highest achievements in discovery, and the best method of public tuition. In other countries, the healing art is divided at every natural seam, and the time is rapidly approaching when our omnibus diplomas will be split up into a number of different charters, for as many different men as they now contain entire professions. The proposition to load them down with another, and an outside passenger now, is forbidden by the whole spirit and policy of progress. As population becomes more dense, and a truer order gets opportunity, a college of surgeons, a college of physicians, a college of obstetricians, and a college of dentists, will be the least simplification that will satisfy, because it is the least that will secure the very best ends; and every one of them will have its special faculty, nearly as numerous as those now charged with the whole round of the healing arts. There is one consideration of pre-eminent importance, if there be any such difference among the several necessary parts of a whole thing, which is almost alone conclusive for the establishment of separate dental colleges; the dispensary and clinic practice which they are capable of affording the pupils, and which cannot be otherwise or elsewhere obtained. No private practitioner can give his students any office work, he cannot turn over his patients to them, even for a small operation, and can therefore afford them no practice whatever, in an art that needs much more skill and culture of hand, eye and manner, than all the other branches of surgery and medicine, put together. But, the dispensary which such colleges, located in large cities, always can supply, will secure the opportunities of as much actual training and experience, with its well graduated responsibilities, as the pecuniary means of the institution can take the expense of; and the aspiring student, of his own liberality and enthusiasm, may purchase opportunities beyond this without stint, and all under the best direction, and with the best assistance. I know not well how to exhibit all the advantages that belong to this feature of the separate system, but it is so obvious to persons in practice, that it will receive ready consideration. The allusions which Dr. Gardette makes to the dental colleges already in existence, are guarded by a delicate avoidance of their "history and influence upon the profession" which personal considerations may commend; but, the notion that their existence is a reproach to the medical schools, that they have been driven into independence by the scorn of the medical profession, and have no other *necessity* for being alive, and that their entire apparatus of men and means might be well and advantageously replaced by a single adjunct professorship in each medical faculty, so far as the doctor's opinion, without its argument, can go, is as severe as it is conclusive; and the colleges existing and their friends, and the colleges in possibility, and their advocates, might well spare the courtesy of the doctor's forbearance, for the sake of hearing the reasons on which his sentence is pronounced. Indeed, persons of a different opinion, cannot help feeling that the writer has taken for *granted* a great many things which nobody can *consent* to grant; and although he is not addressing himself to the question controversially, yet for any purpose in his view, it is quite too much to assume three things so important, as first, the practicability of an additional chair in medical schools; second, the sufficiency of such a chair in such circumstances to the thorough indoctrination, both of physicians, who must extract teeth occasionally in the country, and those students who design the practice of dentistry exclusively, and finally the very broad assumption that a complete faculty is inexpedient and unnecessary to the system of dental education.

I have not paused to press the utter incompetency of the proposed adjunct chair to teach all that can be profitably communicated of dentistry to the pupil, by a system of public lectures, and the incidental means of a college, except on the consideration of certain topics, which bear upon it, for I think it sufficiently disposed of in this way. A closer and fuller inquiry, would demand the anatomy of our profession, or a very thorough analysis of it, and a philosophy of education, besides ; for which there is just now neither time nor necessity. Indeed, I regard the proposition, which I have been discussing with only one apprehension that disturbs me, (I do not suppose that it will take the place, or exclude, or delay the better method,) but I am a little jealous for the reputation of my profession, perhaps a little over sensitive, for I fear some of that contempt which it once endured, both from physicians and the select few of the most distinguished of its own disciples, will get countenance, and a little longer lease of life, from the notion, that a single man in the first rank among us, would willingly cut the connection of the brotherhood, in seeking honor of association with the medical fraternity, that any one of authority among us should esteem a crippling dependency upon medical faculties more creditable and safe, if *not* more useful, than a generous and brave effort at self-adjustment and self-protection. Perhaps, I should add, the possibility of injury to the interests of the profession, from the apparent concession that there is really *nothing* in it which cannot be fully taught by an accommodation chair, in a medical faculty—except, perhaps, the mechanics and pottery of the trade, which the pupil is expected to pick up in any way he can contrive, or if he is not so fortunate, do without, until he has acquired knowledge at the expense of his patients. It is very possible the public may think so, and not a few medical men, simply because they have bestowed little thought on the subject, and there is enough of indifference to professional soundness to allow of a shabby compromise of honor and usefulness for the sake of a dernier resort amongst the chances of practice. Dentistry, in my apprehension, is just now in a critical stage in respect to its system of education. It is just in the condition to demand the sacrifice of all prejudice and selfishness from its most competent and worthiest men, and to need the freest and truest services of all that owe it a duty, or can advance its honor and interests. It would not be in good taste to parade the sentiment which impels me to discharge my debt to the fraternity, or it *might* render me the good service of fully justifying me for "showing my opinion also," and excuse, besides, the uncompromising earnestness of its expression.

